# POU2F1 activity regulates HOXD10 and HOXD11 promoting a proliferative and invasive phenotype in Head and Neck cancer

**DOI:** 10.18632/oncotarget.2492

**Published:** 2014-09-16

**Authors:** Daniel J. Sharpe, Katy S. Orr, Michael Moran, Sharon J. White, Stephen McQuaid, Terence R. Lappin, Alexander Thompson, Jacqueline A. James

**Affiliations:** ^1^ Centre for Cancer Research and Cell Biology, Queen's University Belfast, Belfast; ^2^ Pathology Department, Ninewells Hospital & Medical School, Dundee; ^3^ The Northern Ireland Molecular Pathology Laboratory, Centre for Cancer Research and Cell Biology, Queen's University Belfast, Belfast and The Belfast Trust, Belfast City Hospital, Lisburn Road, Belfast

**Keywords:** Head and Neck cancer, HOXD10, HOXD11, POU2F1, Biomarker

## Abstract

*HOX* genes are master regulators of organ morphogenesis and cell differentiation during embryonic development, and continue to be expressed throughout post-natal life. To test the hypothesis that *HOX* genes are dysregulated in head and neck squamous cell carcinoma (HNSCC) we defined their expression profile, and investigated the function, transcriptional regulation and clinical relevance of a subset of highly expressed *HOXD* genes.

Two *HOXD* genes, *D10* and *D11*, showed strikingly high levels in HNSCC cell lines, patient tumor samples and publicly available datasets. Knockdown of *HOXD10* in HNSCC cells caused decreased proliferation and invasion, whereas knockdown of *HOXD11* reduced only invasion.

POU2F1 consensus sequences were identified in the 5′ DNA of *HOXD10* and *D11*. Knockdown of *POU2F1* significantly reduced expression of *HOXD10* and *D11* and inhibited HNSCC proliferation. Luciferase reporter constructs of the *HOXD10* and *D11* promoters confirmed that POU2F1 consensus binding sites are required for optimal promoter activity.

Utilizing patient tumor samples a significant association was found between immunohistochemical staining of HOXD10 and both the overall and the disease-specific survival, adding further support that HOXD10 is dysregulated in head and neck cancer. Additional studies are now warranted to fully evaluate HOXD10 as a prognostic tool in head and neck cancers.

## INTRODUCTION

Head and Neck cancer encompasses a heterogeneous group of malignancies that can differ markedly in presentation, treatment and prognosis [1]. Approximately 95% of these cancers are squamous cell carcinomas that affect the oral cavity, oropharynx, hypopharynx, nasopharynx and larynx [2]. Head and neck squamous cell carcinoma (HNSCC) accounts for approximately 2% of all malignancies worldwide [3]. Diagnosis of HNSCC is often made at an advanced stage, and despite improved therapeutic regimens over the past few decades, the 5-year relative survival has shown only modest improvement [4]. A better understanding of the pathogenesis of HNSCC may provide useful insights for the development of novel therapeutic strategies.

The *HOX* gene network encodes a family of proteins which act as master regulators of developmental processes. Combinations of *HOX* genes specify the anterior-posterior axis and segment identity during early embryonic development, and postnatally *HOX* genes continue to execute critical regulatory roles in many processes such as apoptosis, receptor signaling, motility and angiogenesis (reviewed by Shah and Sukumar [5]). Numerous observations of dysregulated *HOX* gene expression in solid tumors and leukemia [6] suggest that *HOX* genes are important for both oncogenesis and tumor suppression, but their functional role in cancer onset and maintenance requires further investigation.

There have been relatively few reports of *HOX* gene function in HNSCC, but *HOX* gene expression profiles have been investigated in some related cancers. Takahashi and colleagues analyzed all 39 *HOX* genes by real time quantitative PCR in normal and neoplastic tissue and found altered expression of some genes in thyroid cancer cell lines [7]. Utilizing a similar approach Chen's group found dysregulated expression of *HOX* genes in esophageal squamous cell carcinoma [8] and Hassan and colleagues found that 18 *HOX* genes were significantly higher in oral squamous cell carcinoma than in normal mucosa cell lines [9]. The severely disordered expression affecting multiple *HOX* genes found in these cancers suggests that the normal regulatory processes have become skewed, but to date few transcription factors regulating *HOX* gene expression have been identified [10].

In the present study, we have defined the expression profile of all 39 *HOX* genes in HNSCC cells, the majority of which are upregulated compared to normal oral keratinocytes (NOKs). A subset of highly expressed *HOXD* genes was investigated further by functional knockdown studies and POU2F1 is identified as a transcriptional regulator of both *HOXD10* and *D11*. Detailed examination of a cohort of patient biopsies (n=120) highlights HOXD10 as a potential prognostic biomarker in HNSCC.

## RESULTS

### Occurrence of *HOX* genes in HNSCC cell lines and clinical samples

**Figure 1:**
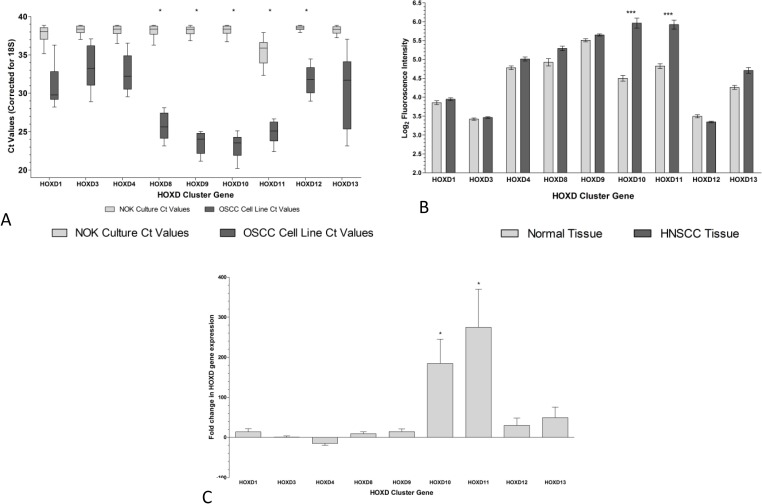
*HOX* genes are highly expressed in Head and Neck squamous cell carcinoma (HNSCC) compared to normal oral keratinocytes (NOK) or control tissue A. Total RNA was extracted from four HNSCC cell lines and three NOK cultures. The expression of each *HOX* gene was analyzed in triplicate. Box plots indicating the range of expression of the *HOXD* cluster in NOKs (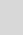), and HNSCCs (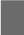) are shown. Whiskers indicate minimum and maximum values; boxes indicate inter-quartile range, with the mean marked. Real-time Q-PCR values were corrected to 18S ribosomal RNA levels. Statistical differences were detected by two-way ANOVA and consistently significant genes are indicated by *. B. Probe intensities of control and tumor tissue were extracted after normalization of expression files. Bars represent mean probe intensity level (±SEM). Significantly different expression was detected by one-way ANOVA, *** p < 0.001. C. RNA was extracted from eight tumor tissue samples and patient matched control tissue. Expression of the HOXD cluster was analyzed by real-time quantitative PCR and the fold difference in expression between matched tumor and control tissue calculated. The mean fold differences (±SEM) are shown and statistical significances were detected by one-sample t-test and are indicated by * (p< 0.05).

Comparative expression profiling by Q-PCR showed that 23 out of 39 *HOX* genes were expressed significantly higher in HNSCCs (n=4) compared with NOKs (n=3) (p<0.05). A striking increase in the expression of four contiguous genes in the *HOXD* cluster (*HOXD8*-*HOXD11*) was evident in HNSCCs (Fig 1A and Supp Fig 1). *HOXD* cluster expression was further analyzed in RNA extracted from a cohort of macro-dissected fresh-frozen tissue samples by Q-PCR. *HOXD10* was 185-fold and *HOXD11* was 275-fold higher in HNSCC tissue compared to the patient-matched control tissue, but none of the other *HOXD* genes were significantly different (Fig 1B).

*HOX* expression was also evaluated in a publicly available microarray dataset comprising 60 HNSCC and 12 control tissue samples. Twelve *HOX* genes showed significantly increased expression in the HNSCC samples, including *HOXD10* and *D11* (Fig 1C and Supp Fig 2), supporting the cell line data.

**Figure 2:**
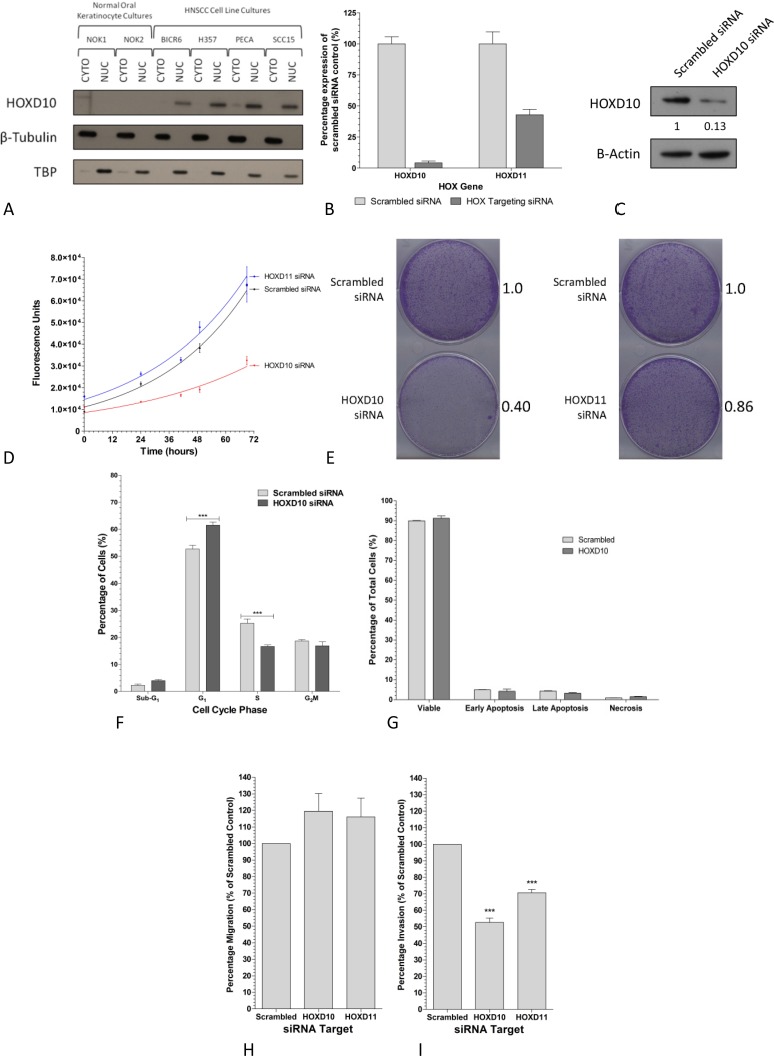
HOXD10 and HOXD11 promote a proliferative and/or invasive phenotype in HNSCC A. Western blot analysis of nuclear and cytoplasmic proteins extracted from HNSCC cell lines and NOK cultures. Appropriate fractionation of the proteins was confirmed by detection of β-tubulin (cytoplasmic protein) and TATA Binding Protein (TBP; nuclear protein). B. Q-PCR analysis confirming depletion of *HOXD10 or HOXD11* expression in HNSCC cells transfected with *HOXD10* or *HOXD11* siRNA. C. Western blot analysis of HOXD10 siRNA transfected HNSCC cells confirming HOXD10 protein depletion. D. The growth of HNSCC cells transfected with scrambled, *HOXD10 or HOXD11* specific siRNAs was assessed using CellTiter-Glo over a period of 70 hours. Graph represents the mean (± SEM) of 3 independent experiments. E. Clonogenic growth assays of HNSCC cells transfected with scrambled, *HOXD10 or HOXD11* specific siRNA. Values represent the mean relative OD540 nm adsorption of three independent experiments normalized to scrambled control F. Cell cycle quantification of HNSCC cells transfected with scrambled siRNA or HOXD10 targeting siRNA by flow cytometry. Statistical differences as determined by two-way ANOVA are indicated by *** (p< 0.001). G. Apoptotic cell populations were detected in siRNA transfected HNSCC cells after 72 hours using annexin V/PI staining and flow cytometry. Graph represents mean apoptotic cell subpopulations as a percentage of total cells (± SEM). H. Quantification of migration of HOX depleted HNSCC cells along a FCS gradient through a porous membrane measured after 24 hours using CellTiter-Glo. Graph represents mean percentage of migratory cells (±SEM) normalized to input cell number expressed relative to scrambled siRNA control. I. Quantification of invasion of HOX depleted HNSCC cells along a FCS gradient through a Matrigel layer measured after 72 hours using CellTiter-Glo. Each assay was normalized to input cell number. Graph represents mean percentage of invasive cells (±SEM) relative to scrambled siRNA control. Statistical differences as determined by one-way ANOVA are indicated by *** (p< 0.001).

Thus *HOXD10* and *D11*, consistently highly expressed in HNSCC cell lines, macro-dissected tumor tissue samples, and in publicly available tissue microarray data from patients with HNSCC, were selected as candidate genes for further study. At the protein level, HOXD10 was shown to be confined to the nucleus, as expected, and higher levels of nuclear HOXD10 were observed in all four HNSCC cell lines compared to the NOK cultures (Fig 2A). No antibody to HOXD11 of adequate specificity for western blotting was commercially available. Targeted knockdown of *HOXD10* or *HOXD11* was confirmed in H357 cells by Q-PCR (Fig 2B) and HOXD10 depletion was confirmed by western blot analysis (Fig 2C). A dramatic decline in the growth rate of H357 cells of approximately 40% was observed after siRNA knockdown of *HOXD10* (Fig 2D) and significant growth inhibition (p<0.001) was further confirmed by crystal violet clonogenic assays compared to scrambled siRNA controls (Fig 2E, left panel). Targeted knockdown of *HOXD11* did not result in significant growth inhibition as determined by the same assays (Fig 2D and Fig 2E, right panel). At the cellular level, a decrease in the rate of cell division in *HOXD10* depleted H357 cells with an increase in G_0_ phase cells and concomitant decrease in the S phase population was demonstrated using propidium iodide staining (Fig 2F). This observed growth reduction does not appear to be due to an increase in apoptosis (Fig 2G). At the functional level, knockdown of either *HOXD10* or *HOXD11* (alone) did not affect H357 cell migration (Fig 2H), however, invasion of the H357 cells through Matrigel was significantly reduced by knockdown of either *HOXD10* or *HOXD11* (Fig 2I). Taken together these results indicate that HOXD10 and, to a lesser extent HOXD11, promote the malignant phenotype of HNSCC.

### Investigation of potential transcriptional regulators of *HOXD10* and *HOX D11*

To identify potential regulators of *HOXD10* and *HOXD11* common to both genes, we searched for transcription factor binding sites (TFBSs) shared by their promoters. We examined 2.5 kb surrounding the start site of all of the *HOXD* cluster genes using MATCH, a weight matrix-based tool for searching putative transcription factor binding sites in DNA [11], with a cut-off to minimize false positive results. The consensus sequences of only 2 transcription factors, namely CUTL1 and POU2F1, were present in the 5′ DNA region of both *HOXD10* and *HOXD11*. POU2F1 consensus sequences were also identified in the 5′ DNA region of *HOXD8* and *HOXD9* which, like *HOXD10* and *HOXD11*, were highly expressed in the HNSCC cell lines.

**Figure 3:**
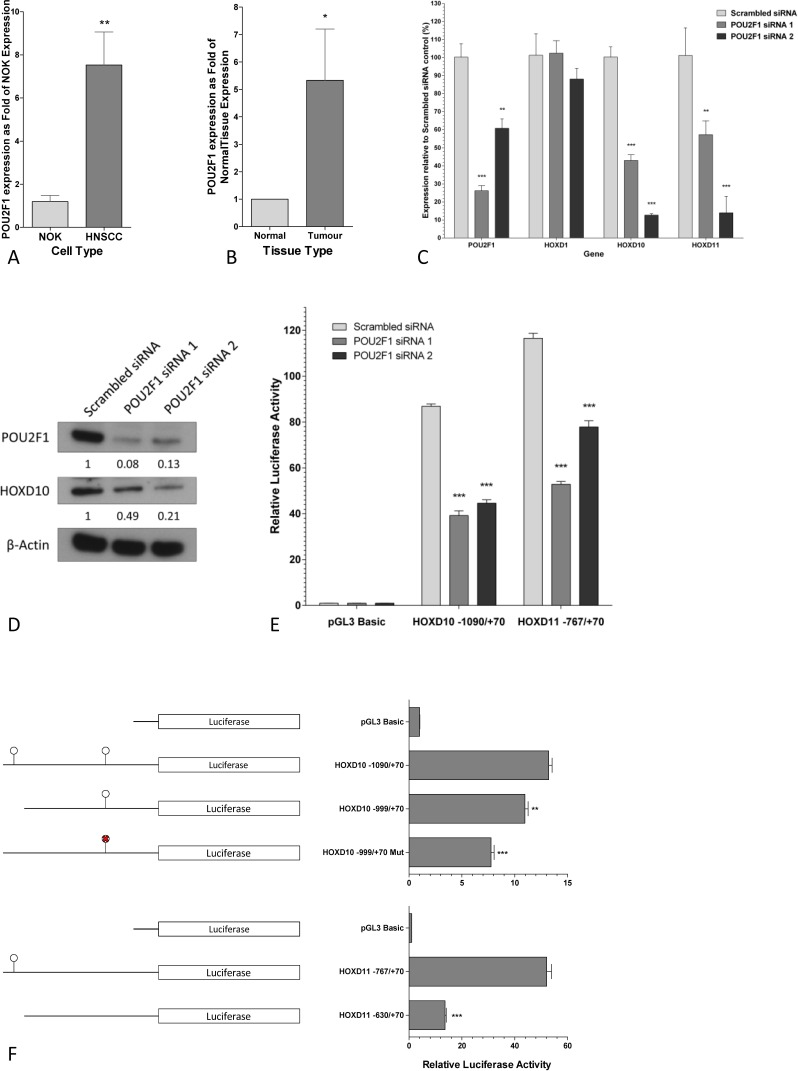
POU2F1 positively regulates the transcription of HOXD10 and HOXD11 A. Q-PCR analysis of POU2F1 in HNSCC cell lines and NOK cultures. The expression of each gene was analyzed in three biological replicates. Graph represents mean fold differences in POU2F1 expression compared to NOK cultures and statistical differences as determined by t-test with Welch's correction are indicated by ** (p<0.01). B. Q-PCR analysis of POU2F1 in HNSCC and normal tissues. Graph represents mean fold differences in POU2F1 expression compared to normal tissue and statistical differences as determined by t-test with Welch's correction are indicated by * (p<0.05). C. Q-PCR analysis of POU2F1 and HOXD gene expression in HNSCC cells transfected with scrambled siRNA or POU2F1 targeting siRNAs 72 hours post transfection. Graph represents mean gene expression levels as percentage of scrambled siRNA control and statistical differences as determined by two-way ANOVA are indicated by ** (p<0.01) or *** (p<0.001). D. Western blot analysis of POU2F1 and HOXD10 expression in POU2F1 depleted HNSCC cells confirms POU2F1 protein depletion and effect on expression of HOXD10. β-Actin was used to confirm equal protein loading. E. Luciferase assays were performed using the proximal promoters of HOXD10 or HOXD11. HNSCC cells were transfected with scrambled siRNA or POU2F1 specific siRNAs and the luciferase activity of the HOXD10 or HOXD11 promoters assessed 72 hours post siRNA transfection. Graph represents the mean normalized luciferase activity (±SEM) and statistical differences as determined by two-way ANOVA are indicated by *** (p<0.001). F. Luciferase assays were performed using promoters of HOXD10 or HOXD11. POU2F1 consensus binding sites are indicated by 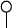. POU2F1 consensus binding sites mutated by site-directed mutagenesis are indicated by 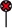. Graph represents the mean normalized luciferase activity (±SEM) and statistical differences as determined by two-way ANOVA are indicated by ** (p<0.01) or *** (p<0.001).

The expression of *POU2F1* was assessed in the four HNSCC and three NOK cell lines, in patient tissues, and in publicly available microarray datasets. In HNSCC cell lines *POU2F1* was 7.5-fold the level in NOKs (p<0.05), as determined by Q-PCR (Fig 3A). In patient tumor tissue samples (n=8) the level of *POU2F1* was higher by 5.3-fold compared to matched control samples (P<0.05) by Q-PCR (Fig 3B). The expression of *POU2F1* was not significantly altered in HNSCC tissue compared to contralateral control tissue in the publicly available microarray datasets (data not shown).

To determine whether POU2F1 acts as a transcriptional regulator of *HOXD10* and *D11* genes, their expression was assessed in *POU2F1* knockdown cells. Decreases in *HOXD10* and *D11* expression of 2- to 8-fold were observed with *POU2F1* knockdown (p<0.01), but there was no change in *HOXD1* level, which does not contain a POU2F1 consensus binding sequence (Fig 3C). The decrease in HOXD10 expression was confirmed by western blot, which showed a 2- to 5-fold decrease in HOXD10 protein after *POU2F1* knockdown (Fig 3D).

Further proof of the regulation of *HOX10* and *D11* by POU2F1 was obtained from luciferase assays using the putative promoter regions containing the POU2F1 binding sequences. When *POU2F1* levels were reduced by siRNA in H357 cells, both the *HOXD10* and *D11* promoter constructs showed significant decreases in activity of approximately 50% (Fig 3E). Deletion of the POU2F1 binding site from the *HOXD10* promoter luciferase construct significantly reduced the *HOXD10* promoter activity by17%. Further analysis of the *HOXD10* 5′ DNA revealed a second, less conserved consensus binding site and mutation of this site further reduced the promoter activity by 24%. Similarly, deletion of the POU2F1 binding sequence in the *HOXD11* promoter significantly reduced the *HOXD11* promoter activity by 74% (Fig 3F). Additionally, ChIP assays confirm direct binding of POU2F1 to the promoter regions of *HOXD10* and *HOXD11* (Supp Fig 3). These data support the contention that POU2F1 is a transcriptional regulator of *HOXD10* and *D11*.

**Figure 4:**
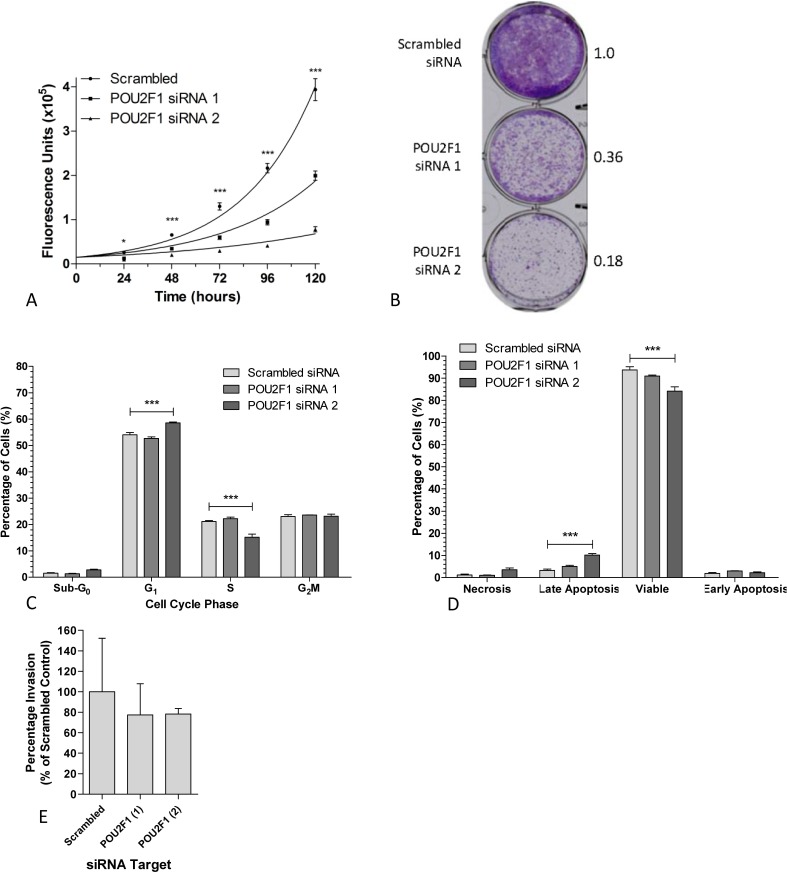
POU2F1 promotes a proliferative phenotype in HNSCC A. The growth of HNSCC cells transfected with scrambled, or *POU2F1* specific siRNAs was assessed using CellTiter-Glo over a period of 120 hours. Graph represents the mean (± SEM) of 3 independent experiments. Statistical differences as determined by two-way ANOVA are indicated by * (p<0.05) or *** (p<0.001). B. Clonogenic growth assays of HNSCC cells transfected with scrambled or *POU2F1* specific siRNAs. Values represent the mean relative OD540 nm adsorption of three independent experiments normalized to scrambled control. C. Cell cycle quantification of HNSCC cells transfected with scrambled siRNA or POU2F1 targeting siRNAs by flow cytometry. Statistical differences as determined by two-way ANOVA are indicated by *** (p< 0.001). D. Apoptotic cell populations were detected in siRNA transfected HNSCC cells after 72 hours using annexin V/PI staining and flow cytometry. Graph represents mean apoptotic cell subpopulations as a percentage of total cells (± SEM). Statistical differences as determined by two-way ANOVA are indicated by *** (p< 0.001). E. Quantification of invasion of POU2F1 knockdown HNSCC cells along a FCS gradient through a Matrigel layer measured after 72 hours using CellTiter-Glo. Each assay was normalized to input cell number. Graph represents mean percentage of invasive cells (±SEM) relative to scrambled siRNA control.

A significant decrease in cell growth was observed upon targeted *POU2F1* knockdown after 48 hr (p<0.001). This effect was much greater with *POU2F1* siRNA 2 than with siRNA 1, equating to increases in doubling time of 116% and 30% respectively (Fig 4A), which was also reflected in a significant reduction (3- to 5-fold) in clonogenic assay cell growth compared to scrambled siRNA controls (Fig 4B). As for *HOXD10*, *POU2F1* siRNA 2 depleted H357 cells showed a significant increase in cells in G_0_ with a concomitant decrease in S phase, whereas siRNA 1 did not elicit any change (Fig 4C). A relatively minor, but significant, increase in the apoptotic cell population was also found with siRNA 2 (Fig 4D). No change in invasive capability was observed after siRNA knockdown of *POU2F1* in H357 cells (Fig 4E).

### Immunohistochemical investigation of *HOXD10* in clinical samples

**Figure 5:**
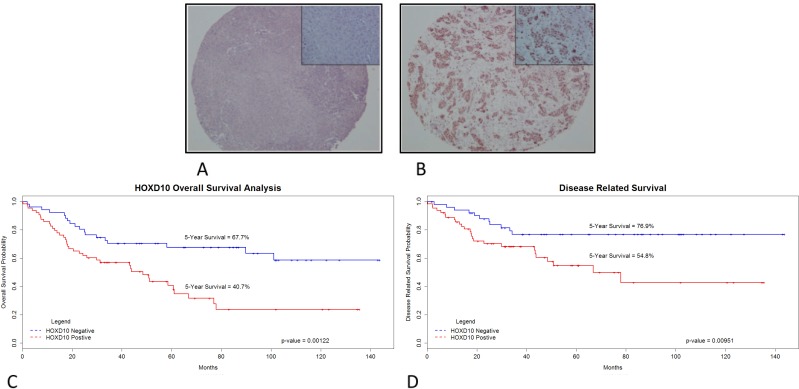
Immunochemical staining of HOXD10 in HNSCC associates with shortened patient survival A. Representative sample of HOXD10 negative (IHC Quickscore< 50) HNSCC patient tissue. Original magnification ×10. Inset magnification x40 B. Representative samples of HOXD10 positive (IHC Quickscore>50) HNSCC patient tissue. HOXD10 immunoreactivity is largely confined to the nucleus. Original magnification ×10. Inset magnification ×40 C. Kaplan-Meier analysis performed using overall survival statistics and HOXD10 immunoreactivity. High HOXD10 expression significantly associated with poor prognosis by the log-rank test (p-value =0.00122). D. Kaplan-Meier analysis performed using disease related survival statistics and HOXD10 immunoreactivity. High HOXD10 expression significantly associated with poor prognosis by the log-rank test (p-value =0.00951).

To examine the potential clinical relevance of HOXD10 in HNSCC, a total of 120 patient samples in a TMA were immunohistochemically stained for HOXD10 protein expression. Six cases were lost to analysis due to insufficient clinical information or tumor representation in the TMA cores. Varying degrees of nuclear staining intensity were observed in HNSCC tissue of the 114 cases available for study (Fig 5A and Fig 5B). The associations between HOXD10 immunohistochemistry positivity and clinicopathological characteristics are summarized in Table 1. HOXD10 staining was significantly associated with increased smoking habit, and with more differentiated and earlier UICC stage tumors. To identify any association with overall or disease-specific survival, Kaplan-Meier survival curves were constructed using HOXD10 immunohistochemical staining to stratify patients (Fig 5C and Fig 5D). A significant difference in the survival curves of HOXD10 positive and HOXD10 negative patients was identified by the log-rank test for both overall (p=0.00122) and disease-specific (p=0.00951) survival. Subsequent univariate Cox proportional hazards models identified significant associations between HOXD10 positivity and reduced overall survival (p=0.00172; hazard ratio = 2.47) and disease-specific survival (p=0.0121; hazard ratio = 2.48), as shown in Table 2. HOXD10 positivity was evaluated for independence from the described clinicopathological characteristics by generating a multivariate model with reverse-stepwise selection of variables. The variables included in the final multivariate models were: HOXD10 positivity, patient age, tumor site and tumor stage. The UICC stage of the disease was also included in the disease-specific survival model (Table 3; Supp Table 1). The association between HOXD10 and overall or disease-specific survival remained independently significant in this model with adjusted hazard ratios of 2.23 (p=0.00916) and 2.62. (p=0.0151) respectively. These data suggest that HOXD10 has a potential role in the prognosis of aggressive HNSCCs and warrants further study as a prognostic tool, independent of the established clinicopathological variables in head and neck cancers.

**Table 1:** Clinicopathological correlations of HNSCC TMA patients with HOXD10 immunochemical staining Correlations between clinicopathological features and HOXD10 immunostaining were assessed by Fisher's Exact Test.

Parameter	Patients N (%)	HOXD10 Negative N (%)	HOXD10 Positive N (%)	Fisher's Exact Test p-value
Age < 57 years ≥ 57 years N/A	59 (51.3) 55(47.8) 1 (0.9)	30 (58.8) 21 (41.2) 0 (0)	29 (46.0) 33 (52.4) 1 (1.6)	0.2567
Gender Male Female	85 (73.9) 30 (26.1)	38 (74.5) 13 (25.5)	46 (73.0) 17 (27.0)	1.0000
Smoking Non-Smoker Light Smoker Moderate Smoker Heavy Smoker N/A	19 (16.5) 8 (7.0) 41 (35.7) 27 (23.5) 20 (17.4)	13 (25.5) 5 (9.8) 12 (23.5) 8 (15.7) 13 (25.5)	6 (9.5) 3 (4.8) 28 (44.4) 19 (30.2) 7 (11.1)	0.0121
Alcohol No Alcohol Light Alcohol Moderate Alcohol Heavy Alcohol N/A	3 (2.6) 21 (18.3) 21 (18.3) 30 (26.1) 40 (34.8)	1 (2.0) 11 (21.6) 7 (13.7) 9 (17.6) 23 (45.1)	2 (3.2) 9 (14.3) 14 (22.2) 21 (33.3) 17 (27.0)	0.2972
Tumour Site Base of Tongue Oropharynx and Pharynx Retromolar Trigone Soft Palate Tonsil	18 (15.7) 14 (12.2) 13 (11.3) 9 (7.8) 61 (53.0)	7 (13.7) 5 (9.8) 2 (3.9) 1 (2.0) 36 (70.6)	10 (15.9) 9 (14.3) 11 (17.5) 8 (12.7) 25 (39.7)	0.0057
Ki67 positive < 35% ≥ 35%	59 (51.3) 56 (48.7)	25 (49.0) 26 (51.0)	33 (52.4) 30 (47.6)	0.8507
Differentiation Well/Moderately Differentiated Poorly Differentiated Undifferentiated/Anaplastic N/A	77 (67.0) 33 (28.7) 0 (0) 5 (4.3)	27 (52.9) 23 (45.1) 0 (0) 1 (2.0)	49 (77.8) 10 (15.9) 0 (0) 4 (6.3)	0.0015
T Stage Stage I Stage II Stage III Stage IV N/A	30 (26.1) 62 (53.9) 14 (12.2) 7 (6.1) 2 (1.7)	11 (21.6) 30 (58.8) 6 (11.8) 3 (5.9) 1 (2.0)	18 (28.6) 32 (50.8) 8 (12.7) 4 (6.3) 1 (1.6)	0.8226
UICC Stage Stage I Stage II Stage III Stage IV N/A	14 (12.2) 15 (13.0) 25 (21.7) 58 (50.4) 3 (2.6)	2 (3.9) 5 (9.8) 11 (21.6) 32 (62.7) 1 (2.0)	11 (17.5) 10 (15.9) 14 (22.2) 26 (41.3) 2 (3.2)	0.0472

**Table 2:** Univariate Cox hazards associated with HOXD10 immuno-positivity and overall or disease-specific survival The correlations between patient characteristics including HOXD10 positivity and survival were assessed using Cox regression analysis.

Parameter	Overall Survival HR (95%CI)/p-value	Disease Related Survival HR (95%CI)/p-value
HOXD10 Negative Positive	REFERENCE 2.47 (1.40-4.36)/1.12×10^-3^	REFERENCE 2.48 (1.22-5.06)/1.21×10^-2^

**Table 3:** Multivariate Cox hazard models associated with overall or disease-specific survival Multivariate Cox models were generated using reverse-stepwise regression to select the independent prognostic variables for HNSCC survival in this patient cohort. The variables included in the final multivariate models were: HOXD10 Immunohistochemical positivity, patient age, tumor site and tumor stage. The UICC stage of the disease was also included in the disease-specific survival model.

Parameter	Overall Survival HR (95%CI)/p-value	Disease Related Survival HR (95%CI)/p-value
HOXD10 Negative Positive	REFERENCE 2.23 (1.22-4.09)/9.16×10^-3^	REFERENCE 2.62 (1.20-5.70)/1.51×10^-2^
Age < 57 years ≥ 57 years	REFERENCE 2.49 (1.43-4.33)/1.28×10^-3^	REFERENCE 2.62 (1.26-5.43)/9.86×10^-3^
Tumour Site Base of Tongue Oropharynx and Pharynx Retromolar Trigone Soft Palate Tonsil	p-value 0.41	p-value 0.011
T Stage Stage I Stage II Stage III Stage IV	REFERENCE 1.28 (0.64-2.56)/0.49 2.36 (0.91-6.08)/0.08 8.71 (3.22-23.55)/1.97×10^-5^	REFERENCE 1.43 (0.31-6.62)/0.65 4.99 (0.98-25.3)/0.05 8.06 (1.44-45.3)/0.02
UICC Stage Stage I Stage II Stage III Stage IV	—	REFERENCE 0.51 (0.06-4.28)/0.53 0.58 (0.09-3.82)/0.57 1.74 (0.29-10.62)/0.55

## DISCUSSION

Definition of the roles of specific *HOX* genes in malignancy is complicated because of functional redundancy in this large family of genes, their transcriptional regulators remain largely unknown, and relatively few HOX target genes have been identified.

In the current study 25 of the 39 *HOX* genes were consistently more highly expressed in HNSCC cell lines than in NOKs. A subset of genes of the *HOXD* cluster, *HOXD8*-*HOXD11*, showed strikingly high levels in HNSCCs compared to the flanking genes in the cluster, and all of the *HOX* genes expressed in the non-malignant cells. Similarly *HOXD10* and *D11* in HNSCC tissue showed increases of greater than two logarithms compared to patient-matched control tissue. The increases in *HOX* expression in HNSCC reflect the results of previous studies of oral squamous carcinoma, esophageal squamous cell carcinoma and thyroid cancer cell lines. In particular *HOXD10* expression was higher in all three studies [7-9] and *HOXD11* was elevated in two of them [7, 9].

In functional studies knockdown of *HOXD10* caused decreased proliferation and invasion, whereas knockdown of *HOXD11* reduced invasion but did not affect proliferation. Knockdown of *HOXD8* or *D9* had no effect on proliferation, invasion or migration. Knockdown of *HOXD10* and *D11,* significantly slowed migration of HNSCC cells through Matrigel. Taken together with the expression data for *HOXD10* and *HOXD11* these results indicate the possibility that the two genes are coordinately regulated.

In order to identify potential regulators of *HOXD10* and *HOXD11* we initiated a search using bioinformatic techniques for transcription factor binding sites in the 5′DNA region of the *HOXD* genes. Two consensus binding sequences, CUTL1 and POU2F1 were identified in the 5′DNA of both genes. Interestingly, POU2F1 consensus binding sites were also identified in the 5′DNA of *HOXD8* and *D9* which were also highly expressed in the HNSCC cell lines. We therefore focused on POU2F1 which belongs to the POU family of transcription factors that control gene expression through interaction with the octamer element 5′-ATGCAAAT-3′ and related motifs.

We found that *POU2F1* is highly expressed in HNSCC cell lines compared to NOKs, and in patient tumor samples compared to tissue-matched control samples. Knockdown of *POU2F1* by siRNA caused significant reduction in expression of *HOXD10* and *HOXD11*, but not *HOXD1*, which does not contain a POU2F1 consensus binding sequence. Upon *POU2F1* knockdown luciferase constructs of *HOXD10* and *D11* showed significantly reduced activity in H357 cells. Reduced activity was also observed after deletion of the POU2F1 binding sites in these constructs. Knockdown of *POU2F1* caused a decrease in the proliferation of HNSCC cells, similar to the effect of *HOXD10* knockdown. However, a complete recapitulation of the *HOXD10* knockdown phenotype was not observed, likely due to the large number of genes known to be regulated by POU2F1 [12-14]. To our knowledge these results indicate for the first time that POU2F1 is an upstream regulator of *HOX* expression, and that it has a role in the development and progression of HNSCC. Among other tissue-specific genes regulated by POU2F1 are *osteopontin* [15], *iNOS* [16] and the caudal homeobox gene *Cdx-2*, itself a transcriptional activator for a cohort of genes specifically expressed in pancreatic islets and intestinal cells and implicated in the prevention of the development of colorectal tumors [17].

In order to define the potential relevance of HOXD10 in patient survival and prognosis we used tissue microarrays in conjunction with immunohistochemical staining. We found a significant association between HOXD10 positivity and reduced overall and disease-specific survival. This association remained significant after adjustment for other clinicopathological variables such as age, tumor stage, smoking and tobacco habit indicating HOXD10 expression is independent of such factors.

Overall our results suggest that *HOXD10* and *HOXD11* act as oncogenes in oral cancer, whereas previous reports have indicated that they act as tumor suppressors. In gastric carcinoma HOXD10 is downregulated and its forced expression is associated with reduced proliferation, invasion, migration and tumor growth [18]. In glioma cells HOXD10 also appears to have a tumor suppressive function as judged by repression of the invasion-related MMP-14 and uPAR genes [19]. In breast carcinoma cell lines loss of HOXD10 expression occurs during malignant transformation and subsequent forced re-expression leads to a more organized, phenotypically ‘normal’ structure in three-dimensional culture [20]. Less is known about the functions of HOXD11 in cancer although it has been identified as a fusion partner with NUP98 in t(2;11)(q31;p15) acute myeloid leukemia [21]. There are few examples of therapeutic agents targeting either *HOX* or *POU* proteins. Several studies using a *HOX*-*PBX* binding inhibitor peptide have shown efficacy at inducing apoptosis in breast, prostate, melanoma and ovarian cancer cells during *in vitro* studies [22-25]. However, the efficacy of the HOX-PBX binding inhibitor peptide in head and neck cancer has yet to be assessed.

In conclusion, the strikingly high relative expression of *HOXD10* and *D11* in HNSCC cell lines, tumor tissue samples, and HOXD10 in the tissue microarray data, combined with the loss of function associated with their targeted knockdown argue for their role as oncogenes in the pathogenesis of HNSCC. Additional studies are warranted to fully evaluate the potential of HOXD10 as a target or prognostic tool in head and neck cancers.

## MATERIALS AND METHODS

### Cell culture

Four HNSCC cell lines were studied (i) H357 (derived from tongue, and received from Professor S. Prime, University of Bristol), (ii) BICR6 (derived from the pharynx, received from Professor K. Parkinson, University of London), (iii) PE/CA and (iv) SCC15 (both derived from tongue and purchased from ATCC). The NOK cultures, which were used between passage 2 and 7, were a gift from Professor C. Irwin, Queen's University Belfast who established the cultures from outgrowths of mucosal samples independently of fibroblast feeder cells (Ethics Ref: ORECNI 06/NIR01/90). The HNSCCs were normally maintained in keratinocyte growth medium (KGM; Invitrogen, Paisley, UK) [26] then switched to NOK media (Epilife supplemented with Human Keratinocyte Growth Supplement; both Invitrogen) prior to experimentation to standardize culture conditions.

### cDNA synthesis and quantitative PCR

Total RNA was isolated from cultured cells using TRIzol (Invitrogen) according to the manufacturer's instructions. RNA from fresh frozen samples of matched control and tumor tissues from patients with HNSCC (n=8) were obtained from the Tayside Tissue Bank (Ethics Ref: TR000105 and TR000114). DNase I (Invitrogen) treated RNA (5 μg) was reverse transcribed using M-MLV reverse transcriptase (Invitrogen) according to the manufacturer's instructions. Reactions were performed in a final volume of 20 μl.

Quantitative real-time RT-PCR (Q-PCR) was performed using TaqMan™ probe-based (Applied Biosystems, Foster City, California) chemistry on the Applied Biosystems 7500 Real Time PCR system to analyze the expression of each *HOX* gene and *POU2F1*. *18S* ribosomal RNA expression was used as an internal standard for normalization. All Q-PCR reactions were performed under the following conditions: 50°C for 2 min, 95°C for 10 min and 40 cycles of 95°C for 15 sec, 60°C for 1 min. The fluorescence was measured during the 60°C step. Primer and probe sequences for each gene target are available on request.

### Western blot

Nuclear and cytoplasmic protein was extracted from cells by suspension in a solution of Buffer A containing 10 mM HEPES pH 7.4, 1.5 mM MgCl_2_, 10 mM NaCl, 0.1% NP-40 and a cocktail of protease inhibitors (Complete Mini Cocktail, Roche Diagnostics Ltd, Lewes, UK). The cells were lysed on ice for 10 min after which they were passed through a 21 gauge needle to ensure complete plasma membrane lysis. Nuclei were pelleted by centrifugation (12,000 x g at 4°C for 2 min) and the supernatant containing cytoplasmic protein was retained. Nuclei were washed in a solution of Buffer A (as specified above). The nuclei were lysed for 10 min on ice in Buffer A and sonicated for 30 sec to completely disrupt the nuclear membrane. Remaining cell debris was pelleted by centrifugation and the supernatant containing nuclear protein retained. Total protein content was determined by the Bradford protein method using the BCA protein assay kit (Pierce, Cramlington, UK). Protein (30 μg) was loaded onto a Tris-Glycine polyacrylamide gel (10%) and subsequently transferred to a nitrocellulose membrane. The antibodies used for Western blotting were β-tubulin (Abcam 1:1000), HOXD10 (Biorbyt 1:200), POU2F1 (Abcam 1:1000) and TATA-BP (Abcam 1:1000).

### RNA Interference

Knockdown of target *HOX* genes or *POU2F1* was performed using pooled siRNAs (*HOXD8* and *HOXD11*, Dharmacon, Waltham, Massachusetts; *HOXD9* and *HOXD10*, Qiagen, Crawley, UK, *POU2F1* siRNA 1, 5′-CCAGCAGCUCACCUAUUAA-3′ and *POU2F1* siRNA 2, 5′-UGAUGCAGAGAACCUCUCA-3′) or control (scrambled, Dharmacon). The HNSCC cell line H357 was seeded at 2 x 10^5^ cells/cm^2^, cultured for 24 hr and transfected with the relevant siRNA (100 nM) using Lipofectamine 2000 transfection reagent (Invitrogen) in serum free media according to the manufacturer's instructions. Four hours post-transfection, 2 volumes of KGM containing 10% FCS were added. After a further 24 hr the transfection procedure was repeated.

### Cell Growth and Proliferation Assays

Transfected cells were harvested after 48 hr, seeded at 1x10^3^cells/well in 96-well plates and allowed to attach for 16 hr. Cell number was assessed at intervals over a 70 hr period using the CyQuant NF Cell Proliferation Kit (Invitrogen). Transfected H357 cells were trypsinized 48 hr post-transfection and seeded in six-well plates at a density of 5x10_3_cells/well. The cells were allowed to grow for 3 days before staining with crystal violet. Crystal violet reabsorption was performed using 0.1 M sodium citrate in 50% ethanol.

### Cell Migration and Invasion Assays

Transfected cells were harvested after 48 hr and migration or invasion assays were carried out using polycarbonate filters (8 mm pore size; Corning, Amsterdam, The Netherlands). Cells in serum-free media were plated into the upper chamber and allowed to migrate along a Fetal Calf Serum (FCS; Invitrogen) concentration gradient for 24 hr. The number of cells migrating to the lower chamber was assessed using the CyQuant Cell Proliferation Kit (Invitrogen). For invasion experiments, the polycarbonate filters were coated in Matrigel (100 μg/cm^2^; BDBiosciences, Oxford, UK) 24 hr prior to the assay, and incubated at room temperature overnight to dry under sterile conditions. The Matrigel was rehydrated with serum-free media 30 min before the addition of cells. Cells were allowed to invade through this layer towards FCS for 72 hr prior to counting.

### Luciferase Assays

HNSCC H357 cells were seeded into six-well plates at a density of 1×10^5^cells/well, transfected with control vector (pGL3-basic empty) or vectors containing approximately 1 kb of 5′ DNA of *HOXD10* or *HOXD11* cloned upstream of firefly luciferase and co-transfected with renilla luciferase. The collection of samples and assays of luciferase activity were performed as previously described [27].

### ChIP assays

ChIP assays were performed as detailed in supplemental methods. Briefly, formalin-fixed chromatin was isolated from H357 and BICR6 cells, sheared by sonication and immunoprecipitated with an anti-POU2F1 antibody. Isolated complexes were washed eight times with RIPA buffer and once with 1x TE before reversal of the DNA-protein crosslinking and DNA purification by QIAquick columns (Qiagen). DNA was subjected to Q-PCR analysis with gene promoter or non-specific region primers to evaluate promoter DNA enrichment.

### Tissue Microarrays and Immunohistochemistry

Tissue microarray sections containing 120 cases of formalin fixed paraffin embedded (FFPE) HNSCC samples were obtained in triplicate from the Northern Ireland Biobank and used to assess the expression of HOXD10. Immunohistochemical (IHC) staining for HOXD10 was performed with a rabbit polyclonal antibody (Biorbyt, orb30360). An initial set of validation experiments was carried out using FFPE sections from human testes to optimize the staining on a fully automated Bond Max Immunostainer (Supplementary methods). Tumor cores (total in triplicate n=360) were scored by two observers (JJ and SMcQ) blinded to the clinical outcomes of the patients. An independent training TMA with 33 HNSCCs (representing oral cavity, oropharyngeal and pharyngeal SCCs) was used initially to establish scoring concordance between observers. Homogeneous staining localized to the nucleus of the tumor cells was scored as positive. In cases of vesicular and open nuclei, the staining pattern was restricted to the nuclear membrane. The intensity of tumor cell staining was scored semi-quantitatively on a four point scale (0 – unstained at high power; 1 – weak; 2- moderate; 3 – strong). A Quickscore was determined for each tumor core and a series of normal tonsil control tissues (Supplementary methods), based on the product of the staining intensity and the proportion of epithelial cells stained positively. Quickscores determined for control tissues were all <50; therefore the score 50 was used to dichotomize the tumor cases into ‘negative staining’ which represented individual cases with an average Quickscore of less than 50, and ‘positive staining’ which represented individual cases with an average Quickscore of 50 or above. Clinical outcomes analyzed included disease-specific survival and overall survival.

### Statistical Analysis

For the *in vitro* tests containing more than two variables statistical analysis was performed by one-way or two-way analysis of variance (ANOVA) followed by Tukey's post-hoc multiple comparison tests. For tests containing only two variables the student's T-test with Welch's correction was used. Statistical significance was taken as p<0.05. The publically available microarray datasets were imported into R/Bioconductor (Version 3.0.0) and normalized by RMA using the package “affy”. Statistical analysis was performed in Graphpad Prism 5.03 after extraction of normalized expression values. Immunohistochemistry data statistical analysis was performed in R/Bioconductor (Version 3.0.0) using the package “Survival”. For each experiment the statistical tests were indicated in the Results sections. Fisher's exact tests were used to analyze associations between IHC scores and clinicopathological features. Statistical significance was calculated at a 95% confidence level. Survival curves were constructed based on the Kaplan-Meier method and compared using Log-Rank tests. For univariate and multivariate survival analysis, the Cox proportional hazard model was employed. The multivariate model was built using a reverse step-wise regression.

## SUPPLEMENTARY MATERIAL FIGURES AND TABLE


