# Supplementary material for: POU2F1 activity regulates HOXD10 and HOXD11 promoting a proliferative and invasive phenotype in Head and Neck cancer
Source: Oncotarget. 2014 Sep 16;5(18):8803–15. doi: 10.18632/oncotarget.2492 (PMC4226723; doi:10.18632/oncotarget.2492)
Supplement: Supplementary file 1 [file oncotarget-05-8803-s001.pdf]

POU2F1 activity regulates HOXD10 and HOXD11 promoting a proliferative and invasive phenotype in head and neck cancer

Supplementary Material

TMA and Immunohistochemistry

To validate the immunohistochemistry methods whole sections from human testes were subject to antigen retrieval for various times in epitope retrieval solutions 1 and 2 before incubation in primary antibody for either 15 or 60 minutes. Detection of antigen-antibody binding sites was with a polymer-based peroxidase system with DAB as substrate. Antigen retrieval in ER1 solution for 20 minutes followed by incubation in primary antibody at 1/100 for 60 minutes was selected as the optimal conditions for HOXD10. Whole face (non-tumour control tonsil tissue) or HNSCC TMA sections were stained using this method. Nuclear localization of HOXD10 was assessed by two independent observers. A Quickscore based on the product of the nuclear staining intensity and the proportion of tumour cells stained positively was calculated for each tumour core. Quickscores were also determined (average of 10 high power fields) for the squamous epithelium on whole sections of 5 normal non cancer tonsil control tissues. The normal control tissues were found to be all <50. This Quickscore for normal tonsils was used to dichotomise the tumour samples into positive (Quickscore >50) and negative (Quickscore <50) for HOXD10 staining.

ChIP Assays

Chromatin was cross-linked using 1.5% formaldehyde in PBS for 15 minutes at room temperature. Cells were collected after two washings with PBS and resuspended in 450 µl collection buffer [100 mmol/L Tris-HCL (pH 9.4) and 100 mmol/L DTT] with protease inhibitors (Complete mini protease inhibitor cocktail tablets; Roche) and incubated on ice for 10 minutes. Cells were collected by centrifugation at 2000 x g at 4°C for 5 mins, washed with PBS and lysed sequentially by resuspension and 5-minute centrifugation at 2000 x g at 4 °C with 1 mL NCP buffer 1 (10 mM EDTA, 0.5 mM EGTA, 10 mM HEPES [pH 6.5], 0.25% Triton X-100), 1 mL NCP buffer 2 (1 mM EDTA, 0.5 mM EGTA, 10 mM HEPES [pH 6.5], 200 mM NaCl) and 1 mL of lysis buffer (10 mM EDTA, 20 mM Tris–HCl [pH 8.1], 0.5% Empigen BB, 1% SDS) was added to each pellet. The samples were sonicated for a total of 40 minutes at high settings using a Diagenode Biorupter with 15-second on/off bursts. The sonicated samples were then centrifuged for 10 minutes at 15000 x g. One percent of the supernatant was taken as input DNA and the remainder of the sample diluted 5-fold in IP buffer (2 mM EDTA, 150 mm NaCl, 20 mM Tris–HCl [pH 8.1], 1% Triton X-100). This was

then subjected to immunoprecipitation overnight with 1 µg specific or IgG control antibodies bound to species/isotype specific magnetic beads (Invitrogen). Precipitated complexes were washed 8 times in RIPA buffer (50 mM Tris–HCl [pH 7.5], 150 mM NaCl, 1% Igepal CA-630, 0.5% Na Deoxycholate, 0.1% SDS) and once in 1X TE. Complexes were removed from the beads through subsequent 15-minute incubations, vortexing, and 5-minute centrifugations with 50 µL of 1% SDS, 0.1 mol/L $NaHCO_3$. Cross-linking was reversed overnight at 65°C; the DNA was purified with QIAquick columns (Qiagen).

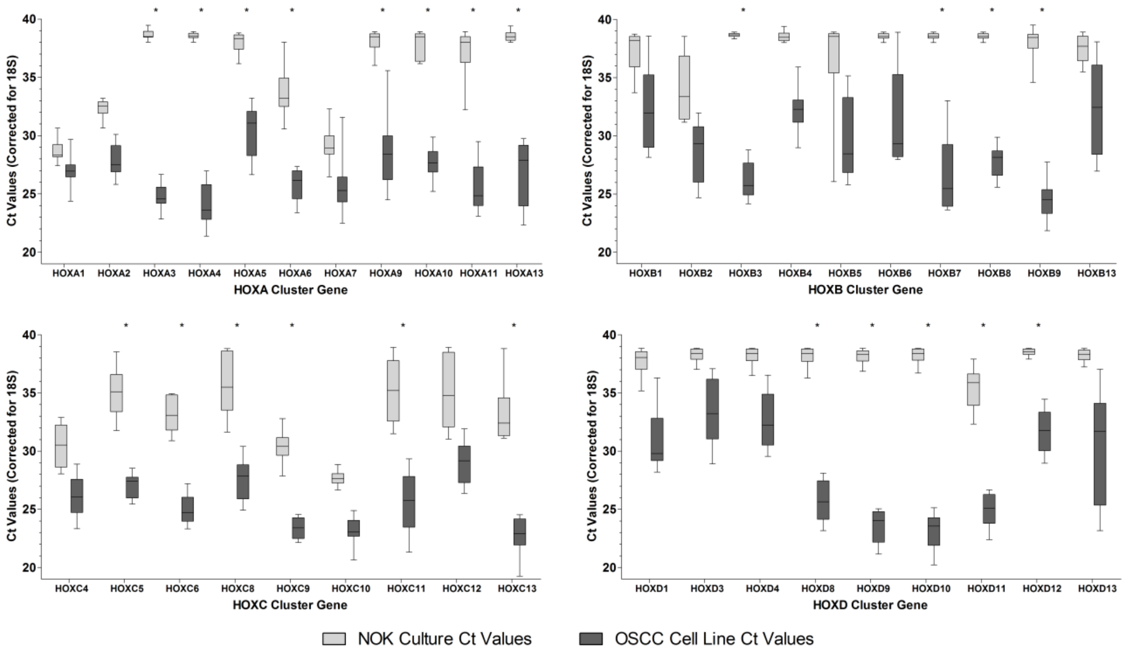

Supplementary Figure 1: Total RNA was extracted from four HNSCC cell lines and three NOK cultures. The expression of each *HOX* gene was analyzed in triplicate. Box plots indicating the range of expression of the *HOXD* cluster in NOKs (▯), and HNSCCs (▮) are shown. Whiskers indicate minimum and maximum values; boxes indicate inter-quartile range, with the mean marked. Real-time Q-PCR values were corrected to 18S ribosomal RNA levels. Statistical differences were detected by two-way ANOVA and consistently significant genes are indicated by *.

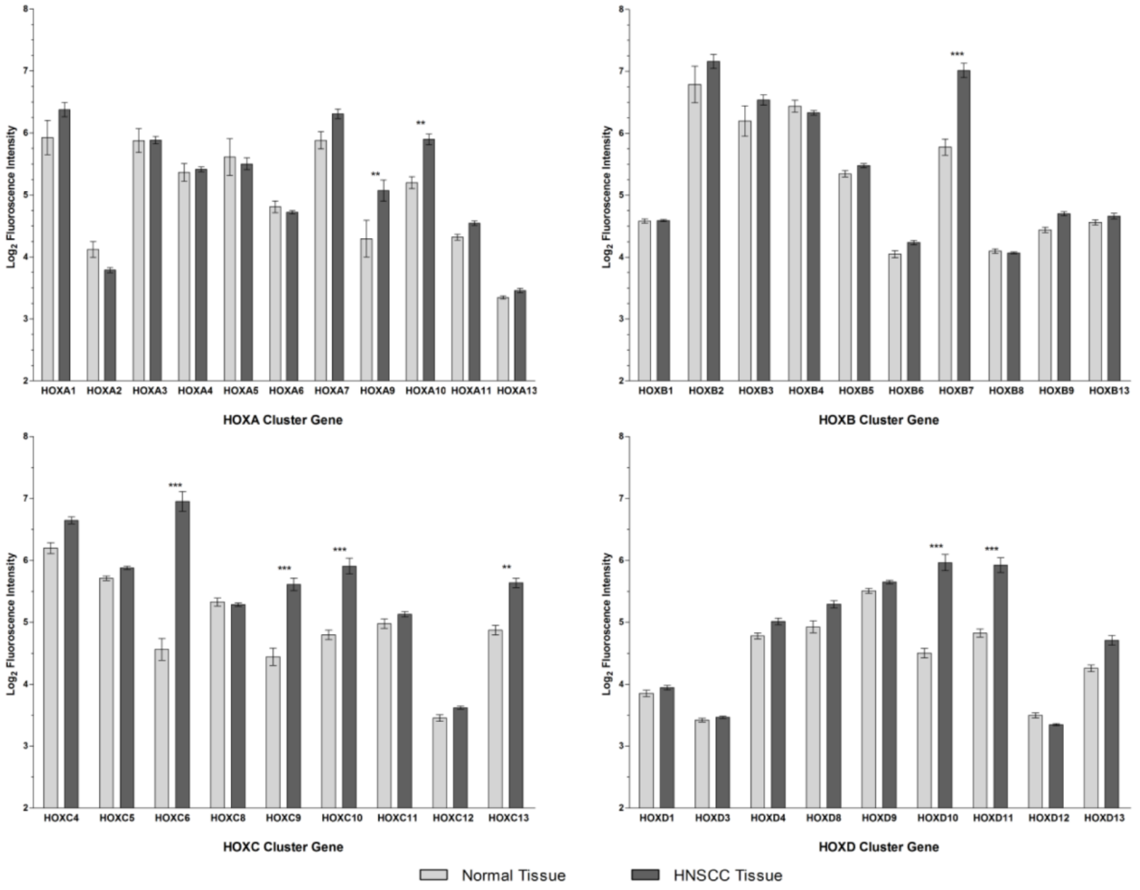

Supplementary Figure 2: Probe intensities of control and tumor tissue were extracted after normalization of expression CEL files in R/Bioconductor. Bars represent mean probe intensity level (±SEM). Significantly different expression was detected by one-way ANOVA, *** $p < 0.001$.

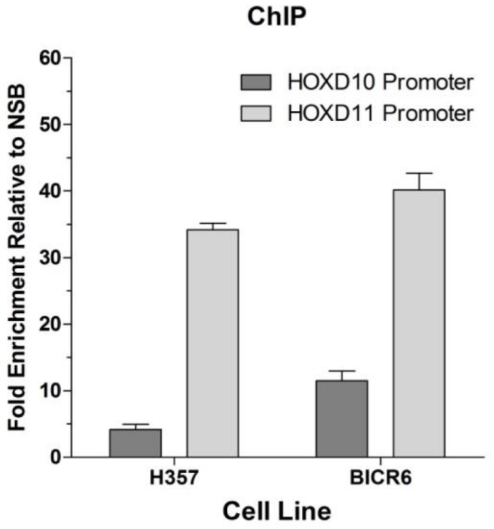

Supplementary Figure 3: ChIP assays were performed from chromatin prepared from H357 and BICR6 cells. Enrichment of POU2F1 binding to HOXD10 or HOXD11 promoter DNA was determined by comparison to a non-specific binding region. The HOXD10 promoter region assessed was -1090 to -999. The HOXD11 promoter region assessed was -767 to -630.

Supp Table 1 : The correlations between HOXD10 immunopostivity, established clinicopathological variables and the overall or disease-specific patient survival were determined using Cox regression analysis. Multivariate Cox models were generated using reverse-stepwise regression to select the independent prognostic variables for overall or disease-specific HNSCC survival in this patient cohort. Statistical significance was calculated for only independently significant variables selected by the reverse-stepwise algorithm. 5-year survival percentages are shown for each variable grouping.

Parameter	5-Year Overall Survival (%)	Overall Survival HR (95%CI)/p-value		5-Year Disease Related Survival (%)	Disease Related Survival HR (95%CI)/p-value	
		Univariate	Multivariate		Univariate	Multivariate
**HOXD10**						
Negative	67.7	REFERENCE	REFERENCE	76.9	REFERENCE	REFERENCE
Positive	40.7	2.47 (1.40-4.36)/1.12×10^{-3}	2.23 (1.22-4.09)/9.16×10^{-3}	54.8	2.48 (1.22-5.06)/1.21×10^{-2}	2.62 (1.20-5.70)/1.51×10^{-2}
**Ki67 Positive Cells**						
< 35%	51.4	REFERENCE	—	69.3	REFERENCE	—
≥ 35%	57.6	0.74 (0.43-1.27)/0.28		61.9	1.19 (0.62-2.27)/0.61	
**Gender**						
Female	55.0	REFERENCE	—	65.0	REFERENCE	—
Male	53.2	1.10 (0.60-2.01)/0.76		65.9	0.83 (0.41-1.68)/0.603	
**Age**						
< 57 years	67.7	REFERENCE	REFERENCE	79.0	REFERENCE	REFERENCE
≥ 57 years	39.4	2.60 (1.50-4.50)/6.65×10^{-3}	2.49 (1.43-4.33)/1.28×10^{-3}	50.5	2.57 (1.31-5.06)/6.34×10^{-3}	2.62 (1.26-5.43)/9.86×10^{-3}
**Smoking**						
Non-Smoker	78.9	REFERENCE		78.9	REFERENCE	
Light Smoker	72.9	1.47 (0.35-6.16)/0.60	—	72.9	1.18 (0.22-6.46)/0.85	—
Moderate Smoker	54.0	1.89 (0.70-5.08)/0.21		73.0	1.42 (0.45-4.46)/0.55	
Heavy Smoker	38.1	3.33 (1.23-8.98)/0.02		46.8	2.91 (0.95-8.94)/0.06	
**Alcohol**						
No Alcohol	66.7	REFERENCE		66.7	REFERENCE	
Light Alcohol	60.3	1.09 (0.14-8.62)/0.94	—	67.6	0.73 (0.09-6.12)/0.78	—
Moderate Alcohol	54.9	0.95 (0.12-7.52)/0.96		71.9	0.56 (0.07-4.85)/0.60	
Heavy Alcohol	59.6	1.21 (0.16-9.22)/0.85		67.2	0.92 (0.12-7.12)/0.93	
**Tumour Site**		p-value 0.17	p-value 0.41		p-value 0.02	p-value 0.011
Base of Tongue	49.4			52.9		
Oropharynx and Pharynx	84.9			42.9		
Retromolar Trigone	62.1			59.8		
Soft Palate	60.0			87.5		
Tonsil	60.5			75.1		
**Differentiation**						
Well/Moderately Differentiated	55.9	REFERENCE	—	66.8	REFERENCE	—
Poorly Differentiated	52.9	0.85 (0.47-1.54)/0.587		60.1	1.07 (0.54-2.13)/0.848	
Undifferentiated/Anaplastic	NA	NA		NA	NA	
**T Stage**						
Stage I	61.2	REFERENCE	REFERENCE	76.1	REFERENCE	REFERENCE
Stage II	59.0	1.26 (0.64-2.47)/0.51	1.28 (0.64-2.56)/0.49	70.5	1.57 (0.62-3.95)/0.34	1.43 (0.31-6.62)/0.65
Stage III	14.7	2.08 (0.85-5.09)/0.11	2.36 (0.91-6.08)/0.08	41.7	4.17 (1.44-12.04)/8.4×10^{-3}	4.99 (0.98-25.3)/0.05
Stage IV	NA	9.29 (3.47-24.84)/9.06×10^{-6}	8.71 (3.22-23.55)/1.97×10^{-5}	NA	11.75 (3.40-40.57)/9.78×10^{-5}	8.06 (1.44-45.3)/0.02
**UICC Stage**						
Stage I	42.4	REFERENCE		66.6	REFERENCE	REFERENCE
Stage II	55.0	0.96 (0.36-2.563)/0.94	—	68.8	0.74 (0.17-3.33)/0.70	0.51 (0.06-4.28)/0.53
Stage III	70.9	0.64 (0.25-1.63)/0.35		78.1	0.98 (0.29-3.34)/0.97	0.58 (0.09-3.82)/0.57
Stage IV	50.1	1.02 (0.47-2.23)/0.96		59.6	1.57 (0.54-4.56)/0.41	1.74 (0.29-10.62)/0.55
